# Focal Ischemic Injury to the Early Neonatal Rat Brain Models Cognitive and Motor Deficits with Associated Histopathological Outcomes Relevant to Human Neonatal Brain Injury

**DOI:** 10.3390/ijms22094740

**Published:** 2021-04-29

**Authors:** Brett J. Kagan, Charlotte M. Ermine, Stefano Frausin, Clare L. Parish, Jess Nithianantharajah, Lachlan H. Thompson

**Affiliations:** The Florey Institute of Neuroscience and Mental Health, University of Melbourne, Melbourne 3010, Australia; charlotte.ermine@florey.edu.au (C.M.E.); Stefano.Frausin@florey.edu.au (S.F.); clare.parish@florey.edu.au (C.L.P.); jess.nithianantharajah@florey.edu.au (J.N.); lachlant@unimelb.edu.au (L.H.T.)

**Keywords:** endothelin-1, cerebral palsy, neonatal stroke, white matter injury, pairwise discrimination, touchscreen testing, rodent models

## Abstract

Neonatal arterial ischemic stroke is one of the more severe birth complications. The injury can result in extensive neurological damage and is robustly associated with later diagnoses of cerebral palsy (CP). An important part of efforts to develop new therapies include the on-going refinement and understanding of animal models that capture relevant clinical features of neonatal brain injury leading to CP. The potent vasoconstrictor peptide, Endothelin-1 (ET-1), has previously been utilised in animal models to reduce local blood flow to levels that mimic ischemic stroke. Our previous work in this area has shown that it is an effective and technically simple approach for modelling ischemic injury at very early neonatal ages, resulting in stable deficits in motor function. Here, we aimed to extend this model to also examine the impact on cognitive function. We show that focal delivery of ET-1 to the cortex of Sprague Dawley rats on postnatal day 0 (P0) resulted in impaired learning in a touchscreen-based test of visual discrimination and correlated with important clinical features of CP including damage to large white matter structures.

## 1. Introduction

Neonatal arterial ischemic stroke is one of the more serious complications of preterm birth [1,2]. Survival rates for preterm infants have increased over time, correlating with an increase in diagnoses of chronic neurological afflictions such as cerebral palsy (CP) [3,4,5,6]. Although tissue damage affects both white and grey matter, it is the extent of white matter injury that has been identified as a critical indicator for CP, where greater damage predicts a less favourable prognosis [3,7,8,9]. There are very few effective treatment options for CP and the need to identify new ones will require animal models that capture key clinical features at both histopathological and functional levels. 

The most widely used approaches to model neonatal brain injury in rodents are based on the protocol originally established by Rice and Vannucci [10] and typically involve some form of arterial occlusion followed by a period of hypoxia. While this approach has been tremendously valuable for our understanding of the pathophysiology of early brain injury, the procedures are technically demanding, often involve a level of mortality and high variability in pathological outcome. It is also a method that has been performed most commonly in rodents around postnatal day 7–10 (P7–10), a time that approximates aspects of human brain development equivalent to near- and full-term infants [10,11,12]. However, earlier neonatal rodent ages between P1 and P5 more closely match pre-term infants based on key developmental hallmarks [13], but present additional technical challenges including this earlier neonatal period being more resistant to damage through conventional hypoxia-ischemia insults [14,15,16].

Recently we reported that intracerebral injection of the potent vasoconstrictor peptide endothelin-1 (ET-1) as a technically simplified model for highly reproducible ischemic damage to the rat brain as early as P0 [17]. This resulted in a stable deficit in motor function persisting into adulthood, along with robust and consistent pathohistological damage characterized by cortical, striatal and subcortical white matter atrophy. Here, we aimed to further develop this model with a focus on assessing impaired cognitive function, a common clinical feature of pre-term brain damage [18].

Compared to motor impairment, robust modelling of cognitive deficits following neonatal brain injury in rodents has been less consistently described (for a review, see [19,20]), and has been dependent on the both the type of cognitive test applied and the model of injury. Our previous study showed that ischemic injury modelled by intra-cerebral injection of ET-1 into neonatal rats at P0 resulted in persistent motor impairment, but no cognitive deficits associated with recognition memory based on a test of novel object recognition [17]. To further explore cognitive impacts in this model, here we used a test for associative learning and memory, the pairwise visual discrimination task using the Bussey–Saksida touchscreen operant system. This method of testing has been found to be highly sensitive to various disruptions, including stress, lesions, and pharmacological interventions [21,22,23]. We report that ET-1 induced ischemia in the early (P0) neonatal rat brain models important features of pre-term clinical brain injury, including significant cognitive and motor impairments with underlying hemispheric atrophy and white matter damage. 

## 2. Results

### 2.1. ET-1 Induced Neonatal Ischemia Results in Deficits in Motor Coordination

**Figure 1:**
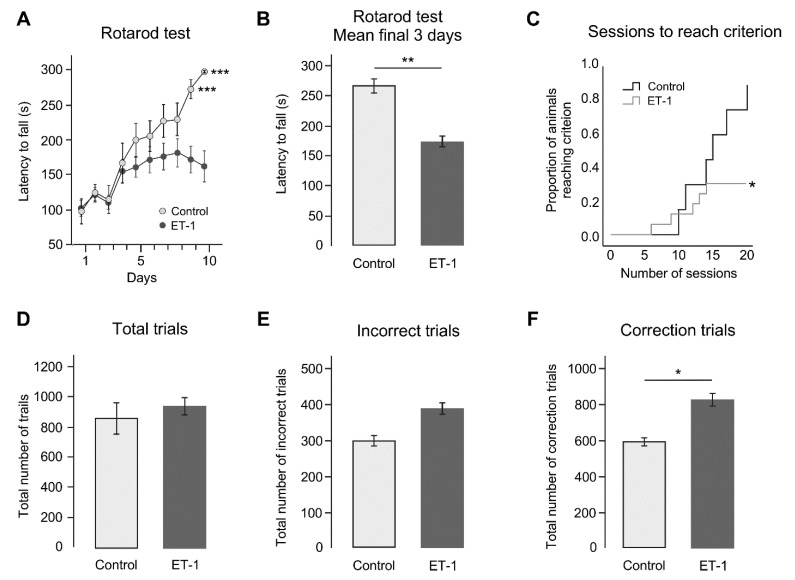
Motor and cognitive changes following early neonatal ischemic injury. (**A**) At 18 weeks of age, latency to fall on the accelerating rotarod over 10 days shows that the control group improves with training to a significantly greater extent compared to ET-1-treated animals. (**B**) Latency to fall on the rotarod represented as average performance over the last 3 days shows the ET-1-treated animals are significantly impaired. (**C**) Proportion of animals reaching the criterion for learning on the pairwise discrimination task over 20 sessions of testing. Less animals in the ET-1 group were able to acquire the learning criterion within 20 sessions. (**D**) Of those animals that did reach the criterion in both control and ET-1 groups, no significant differences could be observed for the total trials taken to reach the criterion. (**E**) Total number of incorrect responses and (**F**) correction trials for all animals tested showed the ET-1 group performed more correction trials than the control group. Error bars = 1 SEM, * *p* < 0.05, ** *p* < 0.01, *** *p* < 0.001, independent *t*-test, (Group size: Control *n* = 7; ET-1 lesion *n* = 17).

Twelve weeks after neonatal delivery of ET-1 or saline, all animals were tested on the accelerating rotarod over 10 consecutive days. The results show that saline control animals improved over this period such that there was a progressive increase in latency to fall, with most animals reaching the test endpoint of 300 s without falling by day 10 (Figure 1A). Animals from the ET-1 group initially improved but plateaued at a significantly lower level of performance that did not improve beyond a latency to fall of approximately 170 s. 

A RM ANOVA was run to compare groups on rotarod latency to fall data (Figure 1A) over 10 days of testing. A significant interaction between time and group was found, F(9, 14) = 2.95, Wilks’ Lambda = 0.35, *p* = 0.034, η_p_^2^ = 0.66, Power = 0.80. The direction of this interaction was followed up focusing on the average latency to fall for both groups across the final three days of testing (Figure 1B) showed that ET-1-treated animals had a significantly impaired capacity to perform (*t*-test for average latency to fall across final 3 days of testing, Control group (266.33 s ± 25.68), ET-1 group (171.85 s ± 80.20), t(22) = 4.35, *p* = 0.006).

### 2.2. ET-1 Induced Neonatal Ischemia Impairs Associative Learning in the Pairwise Visual Discrimination Touchscreen Task

Sixteen weeks after neonatal delivery of ET-1 or saline, animals completed a pairwise discrimination task to evaluate cognitive performance related to associative learning and memory. After 20 sessions of training on the pairwise visual discrimination task, while the majority of animals in the saline group (85.7%) were able to acquire the learning criterion (≥85% correct responses on two consecutive day), approximately a third of the animals in the ET-1-treated group (35.3%) were able to achieve this (Figure 1C). A Kaplan–Meier survival analysis confirmed that there was a significant difference in the proportion of animals in each group reaching the learning criterion (Mantel Cox X^2^ (2, *n* = 24) = 5.16, *p* = 0.023). Of the animals that successfully acquired the pairwise discrimination task and reached the learning criterion, comparison of the total number of trials taken to reach the criterion showed no significant difference between groups (*t*-test; t(22) = 0.73, *p* = 0.473, Figure 1D). Comparing all animals that were tested, although the total number of incorrect responses was greater in the ET-1 group when compared to the control group (Control = 295.86 trials ± 80.48; ET-1 = 384.41 trials ± 134.86; t(22) = 1.61, *p* = 0.122, Figure 1E), this was not statistically different. However, analysis of the total number of correction trials (Figure 1F) showed that the ET-1 group (823.65 correction trials ± 295.74) performed significantly more correction trials compared to the Control group (Control = 587.00 correction trials ± 127.22; ET-1 = 823.65 correction trials ± 295.74; t(22) = 2.74, *p* = 0.012). Collectively, these data indicate that the ET-1 insult robustly impairs associative learning and memory. 

### 2.3. Early Neonatal Ischemia Results in Reduced Adult Cortical and Striatal Volume

**Figure 2:**
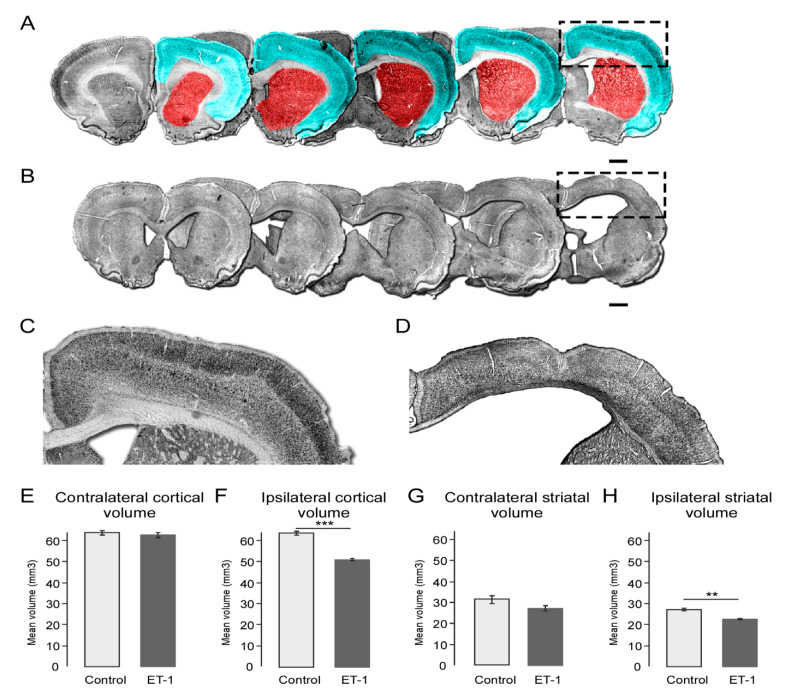
Quantification of cortical and striatal volumes 24 weeks after ischemic injury. (**A**) The cortical (cyan) and striatal (red) regions quantified are illustrated in coronal sections from a representative control animal immunohistochemically labelled for NeuN. (**B**) Immunohistochemistry for NeuN in representative coronal sections spanning the rostro-caudal axis 24 weeks after ET-1 induced cortical ischemia illustrates gross anatomical impact including ventricular enlargement (boxed area enlarged in (**D**)). Representative images of control (**C**) and ET-1 (**D**) cortical areas illustrate the cortical thinning and hydrocephaly resulting from ischemic injury. Quantification of cortical volume showed no significant change between the control and ET-1 groups in the contralateral hemisphere (**E**) but a significantly reduced volume in the ipsilateral hemisphere in ET-1-treated animals (**F**). Similarly, striatal volume was not changed in the contralateral hemisphere (**G**) but was reduced in the ipsilateral hemisphere of animals with neonatal ischemic injury compared to the control group (**H**). Scale bars = 1 mm. Error bars = 1 SEM, ** *p* < 0.01, *** *p* < 0.001, independent *t*-test, (Group size: Control *n* = 7; ET-1 lesion *n* = 17).

At 24 weeks, histological analysis was performed, including measurement of cortical and striatal area in serial sections as shown in Figure 2A. Coronal sections of immunohistochemistry for NeuN in 12 series across the rostro-caudal axis from representative saline (Figure 2A) and ET-1 (Figure 2B) -treated animals illustrate the gross morphological impact of the ischemic injury, including hydrocephaly as well as cortical and striatal atrophy. In addition to thinning, cortical malformation in ET-1-treated animals was characterised by scarring around the injection site and involution reminiscent of ulegyric folding seen in cerebral palsy (Figure 2B,D).

Extrapolation of the area measurements in serial sections was used to estimate cortical and striatal volume. There was no significant change in contralateral cortical size (Figure 2E); however, the ipsilateral cortex was significantly reduced in volume in ET-1- compared to saline-treated animals (Figure 2F; ET-1 = 51.26 mm^3^ ± 4.73, Saline = 64.15 mm^3^ ± 4.77, t(22) = 6.05, *p* < 0.001). Similarly, the contralateral striatal volume was unchanged between groups (Figure 2G) but was significantly reduced in the ipsilateral hemisphere of ET-1-treated animals (Figure 2H; ET-1 = 22.64 mm^3^ ± 2.75, Saline = 27.23 mm^3^ ± 3.43, t(22) = 3.47, *p* = 0.002). 

### 2.4. ET-1 Induced Neonatal Ischemia Results in Specific Patterns of White Matter Damage

**Figure 3:**
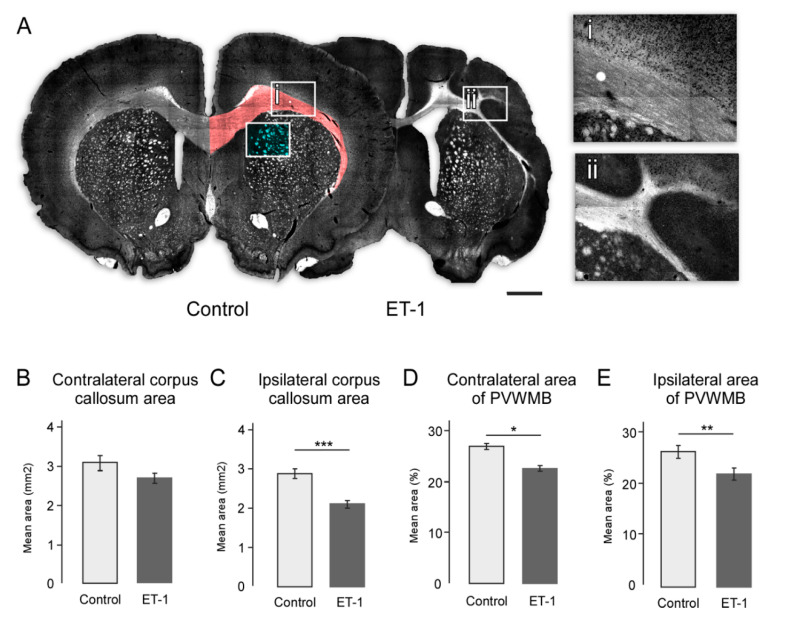
Changes to the size and morphology of major white matter tracts following neonatal ischemia. (**A**) Representative darkfield images of coronal sections from a control and ET-1 group illustrate changes to white matter, with the area used for quantification for corpus callosum (red) and PVWMBs (cyan) indicated and boxed areas enlarged to show normal morphology of the corpus callosum for control animals (**i**) in comparison with the gross morphological changes following neonatal ischemia (**ii**). 20X magnificaiton (**B**,**C**) Quantification of area in coronal sections showed no change in contralateral corpus callosum (**B**) but a significant reduction in size of the ipsilateral corpus callosum (**C**) because of neonatal ischemia. Measurement of the size of the PVWMBs as the fractional contribution of white matter in a fixed field of view showed a significant reduction in PVWMB size in both the contralateral (**D**) and ipsilateral (**E**) hemispheres. Scale bar = 1 mm. Error bars = 1 SEM, * *p* < 0.01, ** *p* < 0.01, *** *p* < 0.001, independent *t*-test, (Group size: Control *n* = 7; ET-1 lesion *n* = 17).

White matter structures including the corpus callosum (CC) and periventricular white matter bundles (PVWMB) were identified by darkfield microscopy (Figure 3A,B). Both structures were affected by ischemic injury and this was particularly conspicuous for the CC ipsilateral to ET-1 injection, which appeared malformed and significantly reduced in size compared to saline injected animals. This was confirmed by area measurement in coronal sections at the level of injection. Both the contralateral and ipsilateral CC area was reduced in size in ET-1-treated animals relative to controls (Figure 3C,D); however, only the difference on the ipsilateral side reached statistical significance (ET-1 = 2.11 mm^2^ ± 0.42, Saline = 2.88 mm^2^ ± 0.34, t(22) = 4.28, *p* < 0.001). The relative size of PVWMBs in each group was measured and represented as the percentage of white matter in a fixed field-of-view in a dorso-medial area of the striatum directly adjacent to the lateral ventricle. This showed that PVWMBs were significantly reduced in ET-1 animals in both the contralateral (Figure 3E; ET-1 = 22.00%, ± 4.79, Saline = 26.20% ± 3.24, t(22) = 0.800, *p* = 0.046) and ipsilateral hemispheres (Figure 3F; ET-1 = 15.59% ± 5.39, Saline = 25.87% ± 3.95, t(22) = 3.13, *p* = 0.005). 

### 2.5. Atrophy Resulting from Neonatal Ischemia Is Associated with Neuronal Loss

**Figure 4:**
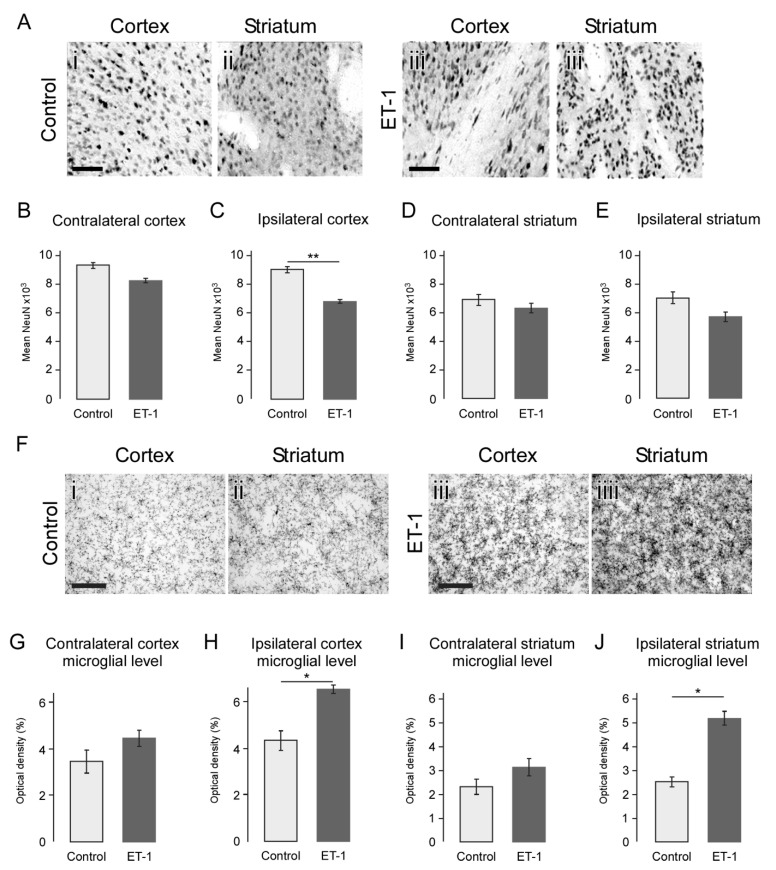
Immunohistochemical assessment of neuronal loss and microglial response to early neonatal ischemic injury. (**A**) Immunohistochemistry for NeuN showing neurons in representative fields of view from cortex and striatum of control and ET-1-treated animals at 24 weeks. Quantification showed that the number of neurons in ET-1 compared to control groups was unchanged in contralateral cortex (**B**) but significantly reduced in the ipsilateral cortex (**C**) and was unchanged in both the contralateral (**D**) and ipsilateral (**E**) striatum. (**F**) Immunohistochemistry for Iba1 showing microglial density in representative fields of view from cortex and striatum of control and ET-1-treated animals at 24 weeks. Quantification showed that microglial density in ET-1 compared to control groups was unchanged in contralateral cortex (**G**) but significantly increased in ipsilateral cortex (**H**) and was also unchanged in contralateral striatum (**I**) and significantly increased in ipsilateral (**J**) striatum. Scale bars: A, C; 100 µm. Error bars = 1 SEM, * *p* < 0.01, ** *p* < 0.001, independent *t*-test, (Group size: Control *n* = 7; ET-1 lesion *n* = 17).

To determine if reduction in tissue volume reflected actual neuronal loss, as opposed to merely a reduction in the extracellular space, neuronal density was measured. This showed that neuronal density was either not significantly different or reduced in the striatum and cortex of ET-1 compared to saline-treated animals (Figure 4A–E) and thus the atrophy was representative of neuronal loss, rather than collapse of extracellular matrix in which case neuronal density would be expected to be increased. The ipsilateral cortex of ischemic animals in fact showed a significant decrease in neuronal density (Figure 4C; ET-1 = 6826.59 NeuN+/mm^2^ ± 1118.49, Saline = 9118.71 NeuN+/mm^2^ ± 1203.81) and thus a loss of neuronal numbers beyond a linear reduction in line with the reduced tissue volume. 

### 2.6. Neonatal Ischemia Results in a Chronic Neuroinflammatory State Persisting up to 24 Weeks

Immunohistochemistry for Iba1 identified microglia as an indicator of inflammation 24 weeks after ET-1 or saline injection (Figure 4F). Inspection of coronal sections showed that early neonatal ischemic injury resulted in a robust inflammatory response that remained at 24 weeks, characterised by a pattern of microglia that appeared much denser in number and with many more cells with an activated, amoeboid morphology, relative to control animals (Figure 4F). Quantification by optical density in fixed fields of view showed that, microglial activation remained significantly elevated in the ET-1-treated animals relative to controls 24 weeks after injury (Figure 4G–J). This was evident in both ipsilateral cortex (ET-1 6.36% ± 1.47, control 4.23% ± 2.25; t(22) = 2.53, *p* = 0.019) and striatum (ET-1 5.16% ± 2.44, control 2.52 ± 1.13; t(22) = 2.71, *p* = 0.013). Although microglial activation also appeared greater in the corresponding contralateral regions, this did not reach statistical significance for cortex (t(22) = 1.58, *p* = 0.129) or striatum (t(22) = 1.36, *p* = 0.189). 

### 2.7. Histopathological Features Correlate with Motor and Cognitive Deficits

To determine whether there were significant correlations between histopathological features of ischemic injury and behavioural outcomes, two-sided Pearson’s correlations were performed. Behavioural measures consisted of rotarod performance as an average of the final 3 days of testing and the percentage of animals to reach criterion in the touchscreen task. Histological measures included cortex and striatal atrophy, size of the corpus callosum and PVWMBs, and neuronal numbers for cortex and striatum. These are presented both for ipsilateral or contralateral hemispheres and summarised in Table 1. 

All significant correlations were associated with damage ipsilateral to the ischemic injury. Significant positive correlations were found between cortical atrophy and both rotarod and touchscreen scores. Similarly, significant positive correlations were found between neuronal density in the cortex and with functional performance in the rotarod and touchscreen tests. Thus, greater neuronal numbers are associated with higher performance across behavioural measures. A significant positive correlation was also found between neuronal numbers in the striatum and touchscreen performance, indicating that greater preservation of striatal neurons is linked with a greater ability to perform pairwise discrimination tasks. Significant positive correlation was also found between CC volume and touchscreen performance. 

### 2.8. White Matter Damage Is a Significant Predictor of Pairwise Discrimination Performance

Given the correlations described above but also taking into consideration the inconsistent capacity for half the animals to meet the basic criterion for learning the pairwise discrimination task, we sought to determine whether any histological variable was a significant predictor of learning. Animals were grouped for the dependent variable based on whether they were considered to have learned the pairwise discrimination task. This yielded two groups, learned (*n* = 12) and not learned (*n* = 12). A discriminant analysis was performed where the key predictive variables were cortical and striatum volume, PVWMB density, and CC area, for measurements both ipsilateral and contralateral to the lesion. Only a single variable, ipsilateral CC area, was found to significantly explain the variance in group member, Wilks’ Lambda = 0.81, χ^2^ = 4.56, F(1, 22) = 5.20, *p* = 0.033. This outcome had an eigenvalue of 0.24 and the single variable explained 19% of the variance in whether animals managed to learn the pairwise discrimination task. This result suggests that the ability to learn a relatively simple pairwise discrimination task is dependent on processes that involve the corpus callosum. 

## 3. Discussion

These results show that local injection of the vasoconstrictor ET-1 into the cortex of early neonatal rats on postnatal day 0 results in behavioural and histopathological features consistent with human prenatal brain injury leading to cerebral palsy (CP). The significant deficits in balance and gross motor coordination measured on the accelerating rotarod are consistent with motor impairments in CP, with clinical spasticity observed in 70–80% of patients, and 10–20% displaying an athetoid or dyskinetic phenotype [24,25]. The stable motor deficit is consistent with multiple studies using the well-established Rice-Vannucci methods of hypoxic-ischemia (HI) in neonatal rodents, typically at the later development stage of P7–P10 [26,27,28,29,30], which approximates certain hallmarks of human brain development equivalent to near or full-term infants [10,11,12]. Here, we show that modelling of cortical ischemic injury as early as P0, which approximates human neurodevelopmental hallmarks closer to pre-term infants [13], also results in significant and chronic gross motor impairment. This is in line with similar findings from our recent work showing stable motor deficit, but no cognitive impairment based on a novel object task after ET-1 induced ischemic injury to the rat striatum at P0 [17].

Here, we have further explored the cognitive impact of early neonatal ischemic injury using a touchscreen approach and show impairment persisting into the chronic phase after injury. While motor function is often prominent and emphasised in disease modelling, assessing cognitive impacts such as deficits in working memory, processing speed and overall intellectual ability are common features of human neonatal ischemic stroke [31,32,33]. In the present study, we detected that ET-1 induced ischemic injury leads to robust impairments in the capacity for associative learning and memory. Not only were animals less likely to reach the learning criterion on the pairwise discrimination task at similar levels to controls, but they made more errors, specifically, correction errors suggesting deficits in the ability to adjust future responding from negative feedback. This is consistent with previous findings using other methods of neonatal injury, including induced hypoxic-ischemic stroke at later neonatal ages, and also other tests, such as the Morris water maze where cognitive function has been found to be impaired [34,35,36]. Notably, in our previous study employing a similar approach resulting in a similar histopathological outcome and gross motor impairment, we did not detect cognitive impairment in a novel object recognition test [17]. This highlights the greater sensitivity of the touchscreen test in the model. It should also be noted, that while only roughly two-thirds of ET-1-treated animals failed to reach criterion, this does not mean that those who did pass were unaffected cognitively. Overall, lesioned animals also performed significantly more correction trials. This suggests that for those who still could reach criterion, significantly more trials were required to get to a similar level of performance and were likely affected by a more subtle deficit. However, this study was not designed to assess specific neural thresholds required in associative learning; however, it forms an interesting topic for future investigations. The finding that some animals in the ET-1 group did reach criterion in the pairwise discrimination test does show a level of variability similar to that which presents clinically in human neonatal ischemic stroke, possibly related to the location and size of the initial ischemic injury.

These functional motor and cognitive impairments were accompanied by a histopathological phenotype that captures important features associated with the diagnosis of CP, including reduced brain volume, cortical malformation, hydrocephaly and damage to major white matter tracts [37,38,39,40]. Moreover, this is commonly observed in HI models, where atrophy occurs ipsilateral to the ligation site [26,27,29]. Quantification of neuronal density showed that the atrophy was indeed associated with neuronal loss, as opposed to collapse of the extra-cellular compartment as we have reported previously for both neonatal [17] and adult [41] models of ET-1 induced ischemic injury in rats. We also observed significant atrophy of white matter structures including the CC and PVWMBs in the dorso-medial striatum; comparable to human clinical data [1,6,25,42,43,44,45,46]. Damage to white matter, particularly axonal injury is also a common clinical observation following neonatal brain damage with evidence of a primary axonopathy [1,6,25,42,43,44,45,46] and has also been captured in previous work in rodents following bilateral carotid artery occlusion in neonatal rats [47,48]. Notably, discriminant analysis of the correlation between histopathological outcomes and functional deficit showed damage to the ipsilateral corpus callosum to have the strongest association with impaired cognitive performance. Such a finding aligns closely with clinical observations that white matter pathology tends to be the most predictive of future cerebral palsy related disability and that the degree of white matter deficits correlates with severity of CP symptoms [46,49,50,51,52].

Another interesting feature of the histological analysis was the chronic nature of a neuroinflammatory state persisting to 24 weeks. Previous work has implicated microglial over-activation in exacerbating secondary brain damage following neonatal ischemic stroke, likely through a combination of oxidative injury, triggering apoptosis, and/or phagocytosis of cells that may potentially recover [53]. This persistent state of chronic microglial activation following a single ischemic event early in life may be an interesting target for therapeutic intervention during the chronic phase after injury. Furthermore, it would be interesting to assess whether this heightened inflammatory state predisposes to an increased pathological response to subsequent injuries. 

In summary, we conclude that focal delivery of ET-1 into the early (P0) neonatal rat brain offers a technically simple approach to model important clinical features of early neonatal brain injury leading to CP. Importantly, this included an impairment of cognitive performance related to associative learning and correlated with damage to white matter. The work also highlights chronic inflammation as an interesting target for therapeutic intervention. It represents a useful model for testing of new therapies for CP both in the acute and chronic phases following neonatal brain injury. 

## 4. Materials and Methods

### 4.1. Animals and Ethics

Ethical approval was granted on the 14 September 2016, by the Florey Institute of Neuroscience and Mental Health Animal Ethics Committee (AEC no. 16-070-FINMH). All experiments were conducted following the guidelines of the National Health and Medical Research Council of Australia Code of Practice and use of Animals for Experimental Purposes in Australia. A total of 24 neonatal Sprague Dawley rats (10 females; 14 males) were obtained from two time-mated dams. At postnatal day 0 (P0) animals were randomly assigned to either a control (vehicle) or an ET-1 ischemia group. Rats were housed with mothers until weaned at 4 weeks when they were then group housed (3–4 rats per cage) in individually ventilated cages. All housing was under standard lighting conditions with a constant ambient temperature of approximately 22 °C. Rats had free access to food and water prior to commencing food restriction for behavioural testing (detailed below). Key variables such as weight and ratio of sex between groups were assessed and found to not differ significantly between groups therefore males and females were treated as one group. 

### 4.2. Surgical Procedures

Rat pups were anesthetised at age P0 through induced hypothermia on wet ice for approximately five minutes and placed in a cooled Cunningham adaptor stage fitted to a stereotaxic apparatus (Kopf, Bergfelden, Germany). A lateral incision exposed the skull in order to locate bregma. An injection of 0.5 µL of ET-1 (*n* = 17, 7 females, 10 males; 400 pmol) or saline vehicle (*n* = 7, 3 females, 4 males) was delivered to the frontal cortex via a micro-syringe fitted with a glass capillary 0.5 anterior and 2.8 lateral to bregma, and 0.5 mm below the dural surface. After injection, the cannula was left in place for five minutes before being withdrawn. The incision was sutured and treated with topical anaesthetic and antiseptic.

### 4.3. Behavioural Testing

**Figure 5:**
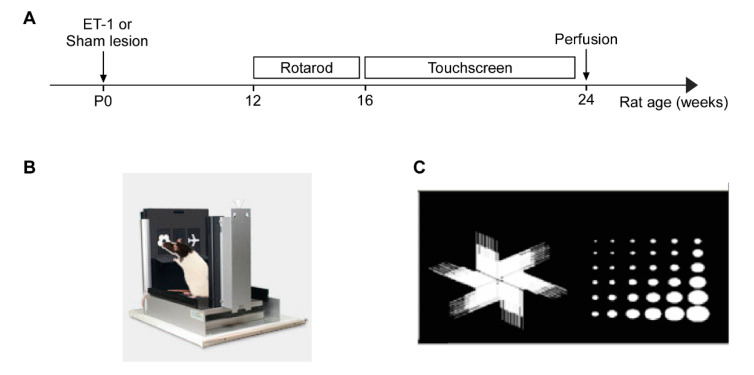
(**A**) Timeline showing time of ET-1 induced neonatal ischemia, behavioural testing and brain tissue collection. (**B**) Representative image of the Bussey-Saksida rat touch screen chamber (**C**) Visual stimuli used in the pairwise discrimination task.

The accelerating rotarod and pairwise discrimination tests were used to assess motor function and aspects of cognitive function, respectively (Figure 5A). 

#### 4.3.1. Accelerating Rotarod

Motor function was assessed at 12 weeks of age, to allow animals to complete most of their natural growth, using the accelerating rotarod. Animals were placed on a rotarod sized for rats (Ugo Basile, Gemonio, Italy) initially rotating at constant speed of 4 rpm and then accelerating to 50 rpm over 300 s. Latency to fall from the rod was assessed twice daily with approximately 45 min between each test over 10 consecutive days. 

#### 4.3.2. Pairwise Discrimination Touchscreen Testing

Cognitive touchscreen testing was assessed starting at 16 weeks of age. For touchscreen testing, animals were food restricted to and maintained at approximately 80–90% of their free-feeding body weight throughout testing. At the conclusion of the touchscreen test, animals were placed back on a free feeding diet. 

Testing was conducted in standard Bussey–Saksida touchscreen chambers for rats (Campden Instruments Ltd. UK/Lafayette Instruments, Lafayette, IN USA, Figure 5B). Testing chambers are trapezoidal in shape (30 cm high × 33 cm long (screen-magazine) × 25 cm wide (at screen) or 13 wide (at magazine) and housed within a dense fibreboard box. The floor is perforated stainless steel raised above a tray. Chambers are equipped with a fan, touchscreen monitor (15.0 inch, screen resolution 1024 × 768), tone generator, house light (LED), magazine unit and pellet dispenser. Following commencing food-restriction, animals were pretrained through 5 stages [23] to train them to make instrumental responses (i.e., nose-pokes) to visual stimuli displayed on the touchscreen in order to obtain a reward. In brief, animals were first habituated to the apparatus (Stage 1) by being placed in the chamber for 30 min a day over two days with reward pellets freely available in the reward magazine. In Stage 2, a visual stimulus is displayed for 30 s on one of two response windows on the touchscreen, and terminated with a tone, reward magazine illumination and the delivery of a reward (one pellet). Next, Stage 3 required animals to touch the stimulus to obtain a reward. Stage 4 extended Stage 3, but now required animals to trigger stimulus presentation referred to as trial initiation. Lastly, the final stage (Stage 5) discouraged animals from touching blank response windows during stimulus presentation by punishing indiscriminate (‘incorrect’) choices. Each pretraining stage required a set performance criterion to be reached before each animal could advance to the next stage as described previously [23]. 

The day immediately following the completion of pretraining, animals were individually advanced onto the Pairwise Discrimination (PD) task, which required learning the association that responses to one of two images (Figure 5C) presented concurrently was correct and thus rewarded, while responses to the other was incorrect and not rewarded. Incorrect responses led to a correction trial, where the previous trial was repeated until a correct response was made. Correction trials were not included in the tally of trials per session. Animals received daily sessions of a maximum of 60 trials or 60 min for 20 sessions, or until animals acquired the learning criterion of ≥85% correct responses (completing 60 trials) on 2 consecutive days. 

### 4.4. Tissue Processing and Histological Assessment

At 24 weeks of age, animals were processed for histological assessment. Following a terminal dose of pentobarbitone (100 mg/kg; Virbac, Peakhurst, Australia) animals were transcardially perfused with 50 mL 0.2 M phosphate buffered saline and 250 mL paraformaldehyde (PFA, 4% in 0.2 M phosphate buffer with 0.2% picric acid). The brains were collected and post-fixed in PFA for 2 h, followed by cryo-protection in 20% sucrose PBS solution for one to two days. The brains were snap frozen on dry ice and coronal sections were collected in series at 40 µm using a freezing-microtome (Leica, Wetzlar, Germany).

Immunohistochemistry was performed on free-floating sections as previously described [54]. Primary antibodies specific for NeuN (1:500; Abcam, ab104225, Cambridge, MA, USA) or Iba1 (1:1000; Wako, West Grove, PA, USA, 019-19741) were incubated overnight to detect neurons or microglial, respectively. After washing, the tissue was incubated with anti-rabbit biotinylated secondary antibody (1:400, Jackson ImmunoResearch, 711-065-152, USA) with 2% donkey serum for 2 h, followed by washing and incubation in avidin-biotin complex (ABC Elite kit, Vectastain; Vector Laboratories, Burlingame, CA, USA) for 1 h. A peroxidase-driven precipitation of diaminobenzidine (DAB) was used to visualise chromogenic labelling, catalysed with a 1% H_2_O_2_ solution and terminated by washing in PBS. 

### 4.5. Histological Quantification

Images were captured using a Leica DM6000 B upright light microscope equipped with a motorised stage to capture whole coronal sections labelled for NeuN. Cortical and striatal area were measured at rostro-caudal levels corresponding to approximately 2.20, 1.30, 0.48, −0.30 and −1.30 mm relative to bregma. The volume of each structure was extrapolated from the cumulative area according to the method of Cavalieri [55]. Quantification of NeuN+ neurons or Iba1+ microglia were performed using a fixed field of view (20× objective) and thresholding of the photomicrographs in ImageJ to produced binarized images. For neurons, total NeuN numbers were counted via the automated function in ImageJ. For microglia, where many of the cells were overlapping, optical density was calculated as the fraction of the field of view occupied by positive signal for Iba1. Darkfield imaging was used to visualise and quantify white matter area, including the corpus callosum (CC) measured as an area and periventricular white matter bundles (PWMB) measured as the fractional contribution of white matter to the fixed field of view (20× objective).

### 4.6. Statistical Analysis

IBM^®^ SPSS^®^ Statistics Version 22 (IBM, Armonk, NY, USA) was used to manage data and run statistical analysis. The descriptive data provided in the text include means and standard deviations, while graphs show means and standard error of the mean (SEM). An alpha of *p* < 0.05 was adopted to establish statistical significance, providing only a 5% chance of a false positive error. Where suitable assumptions were met, independent *T*-tests were run to determine whether statistically significant differences existed between groups. For histological analysis, discriminant analysis was used to predict group membership where the key predictive variables were cortical and striatum volume, PVWMB density, and CC area, for measurements both ipsilateral and contralateral to the lesion. For behavioural analysis, repeated measures analysis of variance (RM ANOVA), *t*-tests or Kaplan–Meier survival analysis were used.

## Figures and Tables

**Table 1:** Pearson’s r correlation coefficients between histological measurements and behavioural assessment scores show a number of significant associations between specific histopathological outcomes and functional deficits. It is interesting to note that these relationships all exist only for histological changes ipsilateral to the ischemic injury, suggesting a lateralisation of impairment that could manifest in measures of motor and cognitive behaviour. * *p* < 0.05, ** *p* < 0.01.

Hemisphere	Measurement	Rotarod	Touchscreen
**Ipsilateral**	Cortical Volume	0.40 *	0.46 *
	Striatal Volume	0.35	0.39
	NeuN density (cortex)	0.55 **	0.48 *
	NeuN density (striatum)	0.36	0.44 *
	Corpus Callosum area	0.20	0.40 *
	PVWMB area	0.32	0.04
**Contralateral**	Cortical Volume	0.22	−0.07
	Striatal Volume	0.35	0.27
	NeuN density (cortex)	0.37	0.32
	NeuN density (striatum)	0.03	0.08
	Corpus Callosum area	0.21	0.33
	PVWMB area	−0.14	−0.29

## Data Availability

Data will be made available by any reasonable request via email to the corresponding author as per MDPI Research Data Policies.

## Abbreviations

CP	Cerebral palsy
ET-1	Endothelin-1
PD	Pairwise discrimination
ITI	Inter-trial interval
IHC	Immunohistochemical
DAB	diaminobenzidine
ABC	avidin-biotin complex
CC	Corpus callosum
PVWMB	periventricular white matter bundles
HI	Hypoxic ischemia
